# Positron Emission Tomography in Breast Cancer

**DOI:** 10.3390/diagnostics5010061

**Published:** 2015-03-16

**Authors:** Jose Luis Vercher-Conejero, Laura Pelegrí-Martinez, Diego Lopez-Aznar, María del Puig Cózar-Santiago

**Affiliations:** 1 Clinical Area of Medical Imaging, Department of Nuclear Medicine, GIBI230, Polytechnic and University Hospital La Fe, Valencia 46026, Spain; 2 Diagnostic Imaging, Sant Joan Despí Moisès Broggi Hospital, Sant Joan Despí, Barcelona 08970, Spain; E-Mail: lpelegri@hotmail.com; 3 Department of Nuclear Medicine, Provincial Hospital Consortium, Castellón de la Plana 12002, Spain; E-Mail: michaeldjjordan@gmail.com; 4 Department of Nuclear Medicine, General University Hospital-ERESA, Valencia 46018, Spain; E-Mail: cozar_mar@gva.es

**Keywords:** FDG-PET, PET/CT, PET/MRI, breast cancer, dedicated breast PET, non-FDG radiopharmaceuticals

## Abstract

Gradually, FDG-PET/CT has been strengthening within the diagnostic algorithms of oncological diseases. In many of these, PET/CT has shown to be useful at different stages of the disease: diagnosis, staging or re-staging, treatment response assessment, and recurrence. Some of the advantages of this imaging modality *versus* CT, MRI, bone scan, mammography, or ultrasound, are based on its great diagnostic capacity since, according to the radiopharmaceutical used, it reflects metabolic changes that often occur before morphological changes and therefore allows us to stage at diagnosis. Moreover, another advantage of this technique is that it allows us to evaluate the whole body so it can be very useful for the detection of distant disease. With regard to breast cancer, FDG-PET/CT has proven to be important when recurrence is suspected or in the evaluation of treatment response. The technological advancement of PET equipment through the development of new detectors and equipment designed specifically for breast imaging, and the development of more specific radiopharmaceuticals for the study of the different biological processes of breast cancer, will allow progress not only in making the diagnosis of the disease at an early stage but also in enabling personalized therapy for patients with breast cancer.

## 1. Introduction

### 1.1. Breast Cancer

Breast cancer is the most common neoplasm in women worldwide and one of the leading causes of cancer-related death in women, with approximately 1.38 million new cases and 458,000 deaths each year around the world [1]. Many risk factors are well-known; however, the exact causes of breast cancer have not been identified [1,2]. Family history of breast cancer, for example, is a well-known factor that increases risk by a factor of two or three. Also, mutations such as BRCA (1 & 2) and p53 are considered to convey a very strong risk of developing the disease [1,2]. Besides these and some environmental risk factors, early detection of breast cancer is considered to be an essential milestone in the control of breast cancer. Early diagnosis is crucial in determining the choice of therapy, as well as a patient’s prognosis and chances for survival [3].

### 1.2. Diagnostic Imaging in Breast Cancer

Anatomic imaging modalities including mammography (MM), ultrasound (US), computed tomography (CT), and magnetic resonance imaging (MRI) are mainly used in clinical practice for detecting primary tumors and staging breast cancer. However, the development of technology in imaging with advanced MRI and CT, and the introduction of other modalities in breast imaging including optical imaging, single photon emission tomography (SPECT), and positron emission tomography (PET) may offer multiple and distinct anatomical, functional, and metabolic information from a macroscopic to a molecular level.

Radionuclide imaging (RI), which includes SPECT and PET, is able to measure *in vivo* cellular, molecular, and biochemical properties of neoplasms and normal tissues. While anatomic imaging modalities focus on improving spatial resolution and image quality, RI offers a more specific targeting of breast cancer with a greater contrast between tumor and normal tissue.

By imaging specific biological processes, RI gives one step forward in cancer detection in addition to anatomical imaging techniques. Variations in phenotypes and biochemical changes in tissues have emerged as especially relevant new targets [4].

RI uses radionuclides combined with other elements to form chemical compounds, or else combines with existing pharmaceutical compounds to form what is called radiopharmaceuticals or radiotracers.

These radiopharmaceuticals can radiolabel specific targets by labeling molecules (^18^F-FDG, ^18^F-FLT, ^18^F-MISO, ^18^F-NaF, ^11^C-choline, ^11^C-acetate), proteins (^18^F-annexin V, ^64^Cu-DOTA-VEGF121, ^99m^Tc-VEGF), antibodies (^99m^Tc-rituximab), chelates (^99m^Tc-setamibi, ^99m^Tc-HDP), and cellular receptors (^18^F-FES, ^68^Ga-DOTATOC), or even act directly as ions (^99m^Tc-pertechnetate, ^123^I-sodium iodine) enabling the evaluation of biochemical changes, cellular physiology, cellular function and metabolism, and levels of molecular targets in an individual.

Through the years, advances in imaging technologies and radiochemistry have been used to develop new radiolabeled molecules to target different tissues, organs, or even molecules rather than just anatomy.

SPECT imaging uses nuclides such as ^99m^Tc, ^123^I, and ^111^In, among others, that decay while emitting single γ-ray photons with different energies. A gamma camera rotates around the patient, acquiring data from different positions, and then tomographic reconstruction is applied. However, unlike positrons emitted by PET agents, γ-ray photons do not deliver adequate information regarding the origin of the photon, making it impossible to define the line of response, as determined in PET technology [5]. In order to address this issue, collimators made of lead or tungsten are used to discard any diagonally incident photons, but, on the other hand, the collimators will exclude those photons that will not properly reach the crystal of the detectors, affecting the sensitivity of the system [5] compared to PET technology.

The most commonly used radiotracer that has been used in SPECT imaging for breast imaging is ^99m^Tc-sestamibi (MIBI), a single-photon radiopharmaceutical. MIBI scintigraphy is a functional imaging study introduced in the early 1990s, now mainly used for cardiac imaging. First described by Aktolun *et al.* [6], and reported in breast cancer by Campeau *et al.* [7], MIBI enters the cell through passive movement from the extracellular compartment to the cytoplasm and accumulates in the mitochondria. Most malignant cells have higher intracellular mitochondrial concentration and thus MIBI accumulation will concentrate in tissue in proportion to that activity [8].

Brem *et al.* [9] studied the sensitivity and specificity of MIBI for the detection of breast cancer and compared the gamma imaging findings with histopathology. One hundred forty-six women were retrospectively studied, with 167 lesions biopsied. Authors claimed a sensitivity of 96% in detecting breast cancer in 80 out of 83 malignant-proven lesions, but showed a moderate specificity (59%). They concluded that MIBI imaging could help detect breast cancers.

In recent years, high-resolution gamma cameras, specific to individual organs, have been developed, in particular for breast molecular imaging [9,10]. These devices seem to improve the overall performance, as will be discussed in Section 3.

Contrary to SPECT agents, PET agents use pharmaceuticals labeled with positron-emitting radionuclides, produced mainly by particle accelerators also called cyclotrons. Some advantages of PET are based on the very high sensitivity and quantitative capabilities, presenting a broad impact in the clinical field, particularly in oncology. The innumerable possibilities for targeting a specific molecule, receptor, antibody, or drugs make RI a unique tool for clinical, pre-clinical, and research studies.

Generally, malignant cells have enhanced glucose metabolism due to accelerated tumor growth as well as an increase in glucose transporter proteins compared to non-malignant cells, and thus an increased glycolytic activity [11]. This high glycolytic activity eases the detection of malignant cells using FDG-PET imaging. Unfortunately, ^18^F-fluorodeoxyglucose (FDG) is not a cancer-specific tracer since it has an increased uptake in inflammatory and infectious lesions and even a significant number of physiologic processes such as brain glucose uptake or muscle uptake [12,13,14].

Although many radiotracers have been developed for PET imaging, most breast cancer imaging studies have been performed with FDG [15]. FDG is a glucose analog transported via glucose transporters into the cells, phosphorylated by hexokinase. FDG mimics glucose during the first enzymatic reactions in the cells, but because FDG lacks a hydroxyl group at the C-2 position, further catabolism of this radiotracer will not be possible. FDG becomes metabolically trapped in tumor cells at a rate proportional to glucose utilization and therefore glucose metabolism [16].

Some authors have demonstrated certain correlation between the degree of FDG uptake and several phenotypic features including tumor histologic type and grade, cell receptor expression, and cellular proliferations index [17,18]. Avril *et al.* [17] quantified FDG uptake in breast tumors and compared it with the histologic type, grading, and some other parameters in 50 invasive and six non-invasive breast carcinomas, finding a positive correlation mainly between the uptake of FDG and the histologic tumor type, microscopic tumor growth pattern, and tumor cell proliferation. However, they did not find a significant relationship between FDG uptake and other factors such as tumor size, steroid receptor status, and expression of the glucose transporter protein GLUT-1, concluding that though some positive correlations were observed, the degree of metabolic changes after a malignant transformation was most likely explained by a number of complex interactions between the need for energy of malignant cells and their tumoral microenvironment showing some limitations of FDG-PET imaging. In another study, Bos *et al.* [18] studied the relationship between FDG and some biomarkers known to be involved in underlying biologic mechanism in FDG-PET scans of 55 breast cancer patients. In this case, the authors found a positive correlation between FDG uptake and GLUT-1 expression, the mitotic activity index, the number of tumor cells, expression of hexokinase type I, and microvessel density, concluding that FDG uptake in breast cancer could be related to the microvessel density, GLUT-1 expression, activity of hexokinase-I, and proliferation rate, among other factors.

Many studies have proven the excellent utility of PET in detection, staging, radiotherapy planning, and treatment response assessment in many oncological diseases. PET has proven to be a very useful tool in staging of advanced breast cancer and in assessing response to therapy widely used in clinical care. These benefits will be discussed in the following section.

PET systems, most of them in their hybrid modality with CT (PET/CT), are mainly designed for whole body imaging, this being one of the strengths of this modality. However, in order to improve the spatial resolution for certain organs such as the breast, new organ-specific PET systems have been developed demonstrating better diagnostic accuracy in primary tumors or suspicion of recurrence than whole-body PET devices. These breast-based imaging modality systems are commonly named Positron Emission Mammography or PEM and will be later discussed.

## 2. Clinical Applications

Many studies have shown high sensitivity and specificity of FDG-PET for the detection of primary large and palpable breast tumors [19,20]. Nevertheless, the sensitivity decreases when the lesions are small and non-palpable, low-grade, or non-invasive neoplasms [20].

As already stated, FDG-PET has been used in breast cancer for diagnosis, staging and re-staging, and treatment response evaluation. However, results presented by several groups have shown a wide variety of conclusions for the use of PET in breast cancer [21,22,23,24,25,26,27,28]. In addition, there are some limitations and pitfalls that may lead to false-positive or false-negative results when assessing breast diseases.

FDG uptake is observed in a variety of benign entities and in several physiologic conditions. FDG may accumulate in areas affected by various non-neoplastic conditions, for example in acute and chronic infections or inflammations and non-specific uptake in brown adipose tissue [29,30]. This increased FDG uptake is produced by the increase of glycolytic metabolism because of the leukocytic infiltration that occurs during inflammatory events. Hence, this can be seen in patients who recently had surgery or radiation therapy. To correctly interpret these findings, the reader should be familiar with the normal physiologic distribution of the tracer, the physiologic variants, and benign causes that show an abnormal FDG uptake. These situations may result in false-positive findings and therefore be confused with malignant processes.

On the contrary, small tumor size (depending on the spatial resolution of each system) or less aggressive histologic subtypes may be the cause for false-negative results [31,32,33]. Kumar *et al.* [33] studied 111 patients (116 breast lesions) with known or suspected breast cancer. An FDG-PET scan was performed for staging purposes and PET results were correlated with follow-up surgical pathology results. Of the 85 pathology-proven malignant lesions, 44 were false negative in PET. The authors found a significant difference in the tumor size, when the tumors were ≤10 mm (*p* = 0.003), and low tumor grade (*p* = 0.001) in patients with true positive and false negative results in PET. They concluded that tumor size and tumor grade were independent factors that predict FDG-PET results.

Some authors have stated that dedicated breast PET devices or a breast-positioning device for PET may improve detection of breast cancer and therefore reduce false-negative results. Kaida *et al.* [34] investigated the detection rate of breast cancer in 660 healthy women that underwent both a conventional whole-body PET study and breast PET imaging in prone position using the breast positioning device. Of the 660 individuals, seven showed breast cancer lesions using the breast-positioning device in PET, but only five of these were positive by whole-body PET study. The authors concluded that prone breast imaging using a positioning device might help to improve the detection rate in breast cancer patients. Dedicated breast PET devices will be discussed in Section 3.

The role of PET and PET/CT in the different phases of breast cancer is discussed in detail.

### 2.1. Diagnosis of Primary Tumors

To date, MM is the standard of reference for the detection of primary tumors, being the only screening method that has proven to have a significant influence on patient survival. Therefore, early detection of the disease is the most effective method for reducing mortality. Either MM or ultrasound (US) detects the changes in the anatomy of the breast tissue; nonetheless, metabolic changes occur before anatomical changes. The accelerated metabolic activity in oncological cells is the key point when thinking about the usefulness of FDG-PET imaging for detection of malignant tumors.

Some researchers have shown very promising results for FDG-PET, with a sensitivity of 80%–96% and specificity of 90%–100% [35,36,37]. In addition, the sensitivity in differentiating benign from malignant tissue is approximately 90% in most of the studies. These results demonstrate that the capabilities of PET are limited by the size of the lesions (around 1 cm) and therefore affect its spatial resolution. However, the constant development of this technology has led to improvements, enabling detection of lesions less than 1 cm, even down to 0.3–0.5 cm in size in some PET systems.

A known limitation of PET in breast pathology is the histological type and grade of the tumoral cells being less sensitive for the detection of invasive lobular carcinoma and slow-growing cancers like tubular carcinoma than for invasive ductal carcinoma [31,35,36,37,38,39]. Heudel *et al.* [38] analyzed the correlation between standard uptake value (SUV) and histopathological and immunohistochemical prognostic factors in 45 women with biopsy-proven primary breast cancer. A positive relationship was found between FDG uptake and histological grade (*p* < 0.0001), histological type (*p* = 0.001), tumor size (*p* < 0.0435), estrogen receptor status (*p* < 0.0005), and progesterone receptor status (*p* = 0.002). The authors demonstrated that these known prognostic factors in breast cancer correlated well with the SUV, a noninvasive metabolic parameter. In another study, Ekmekcioglu *et al.* [39] also investigated the prognostic value of FDG-PET uptake in breast carcinomas by comparing it with histopathological and immunohistochemical prognostic factors (tumor size, histological type, histological grade, pleomorphism, mitosis count, lymphatic invasion, necrosis, estrogen negativity, Ki-67 level, axillary lymph node involvement, and triple negativity). The investigators showed that only a high Ki-67 level and tumor size were determining factors for high FDG uptake values, concluding that FDG uptake may serve as a prognosis indicator for biological behavior in breast tumors.

The majority of the studies published show diagnostic performance results based on a PET scan alone; however, it has been confirmed that specificity obtained with the use of PET/CT significantly improves these results. In one study where PET, CT, and PET/CT were compared in breast cancer patients [40], the diagnostic confidence of PET/CT was higher than with PET or CT alone in more than 50% of the patients studied.

Some other studies do not support the use of FDG-PET as a diagnostic tool [26]. Fletcher *et al.* [41] did not recommend its use for screening purposes but only when tumors were bigger than 2 cm, of aggressive types, or rising tumor biomarkers were present.

### 2.2. Staging and Re-Staging

One of the most important prognostic factors in breast cancer is the status of the axillary lymph nodes. Axillary lymph node dissection is the best approach for evaluating infiltration at this level; nevertheless, morbidity including lymphedema and significant costs are associated with this procedure. Sentinel lymph node biopsy (SLNB) using either dye contrast material or preferably radioisotopes has been accepted as the procedure of choice for nodal assessment. However, this practice still requires surgery and therefore the potential complications that surgery implies.

An alternative to this invasive technique could be FDG-PET to predict lymph node metastasis from breast cancer [21,23,42]. One of the advantages of PET is the ability to detect malignant pathology due to an abnormal increase of the glucose metabolism even in non-pathologically enlarged lymph nodes not captured by CT.

Several authors have presented high sensitivity and specificity rates in the evaluation of axillary lymph nodes using FDG-PET [31,43,44]; however, this seems to be insufficiently accurate to replace the already accepted techniques for axillary staging. Some metastases can be microscopic and because of the limited spatial resolution could be a reason for false-negative in FDG-PET [31,32]. Zhang *et al.* [32] studied the diagnostic accuracy of FDG-PET/CT in detecting primary invasive breast cancer, including invasive ductal breast cancer, invasive lobular breast cancer, and axillary, internal mammary, and supraclavicular lymph nodes metastases. One hundred sixty-four women with operable invasive breast cancer and clinically negative lymph nodes underwent FDG-PET/CT and were retrospectively analyzed and compared with the histopathology of dissected axillary lymph nodes. The diagnostic performance of FDG-PET/CT was significantly correlated with primary tumor grade (*p* = 0.003) and size (*p* = 0.0007). However, the sensitivity, specificity, overall accuracy, positive predictive value, and negative predictive value in the staging of axillary lymph nodes dissection were 46.3%, 91.1%, 79.8%, 63.3%, and 83.6%, respectively, with a false negative rate of 54% mainly because of micrometastases. The investigators concluded that FDG-PET/CT was helpful in the detection of the primary breast cancer and distant metastases but had a limited value in the axillary, internal mammary and supraclavicular lymph nodes evaluation.

Most recently, Riegger *et al.* [45] evaluated FDG-PET/CT in comparison to US as the standard of reference of non-invasive imaging techniques in the detection and accuracy of axillary lymph node metastases. The authors determined that though FDG-PET/CT seems to be more accurate than US, it is only as sensitive as US in the detection of axillary lymph node metastases and therefore it is not recommended as a substitute for SLNB. On the contrary, FDG-PET/CT was able to detect previously unsuspected loco-regional extra-axillary lymph node metastases [45]. Aukema *et al.* [46] also supported the usefulness of FDG-PET/CT as an additional imaging tool to assess extra-axillary lymph node metastases with a significant impact on the management of the patients. This shows the potential advantage of PET/CT found in the evaluation of regional lymph nodes in particular locations such as in the internal mammary and supraclavicular lymph nodes.

**Figure 1:**
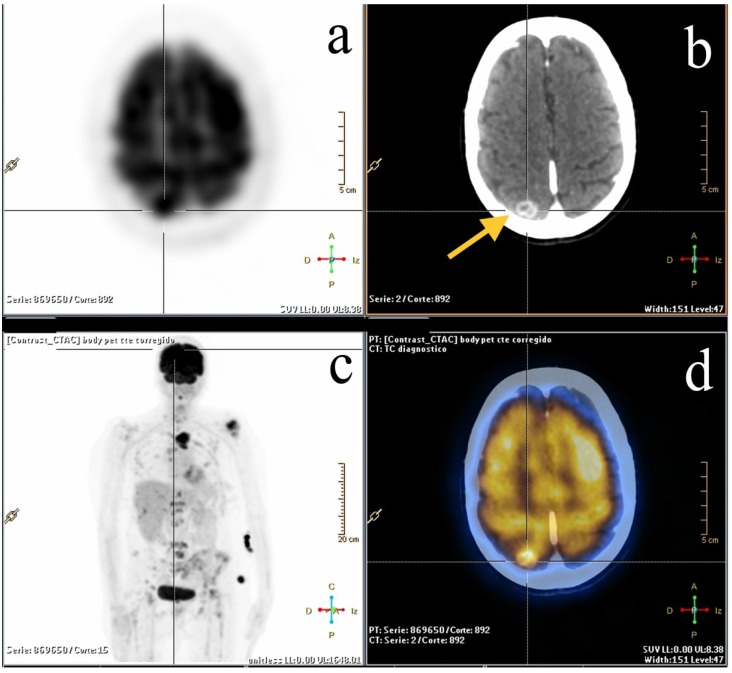
A 67-year-old woman with breast carcinoma and known bone metastases, but without suspicion of progression, referred for FDG-PET/CT exam. (**a**) Axial PET image showing a focus of FDG uptake in the right posterior parietal lobe; (**b**) axial CT showing a rounded lesion with contrast enhancement ring and perilesional edema (arrow); (**c**) MIP image demonstrating the known multiple bone metastases; (**d**) fused image showing a rounded hypermetabolic new lesion consistent with brain metastases.

It is well known that the prevalence of distant metastases is directly related to the staging of the disease at diagnosis [21,23,42,46,47]. As the staging of the disease increases, the possibility of having distant metastases also increases. Probably the most important benefit of FDG-PET/CT compared with other imaging modalities is the capability of detecting unknown distant metastases in a single whole-body study (Figure 1) [31]. Sensitivity and specificity in detecting distant metastases are very high, in the range of 86%–100% and 90%–98% respectively [31,48,49]. Dirisamer *et al.* [49] evaluated the sensitivity and specificity of contrast-enhanced CT (ceCT) and PET/CT for re-staging of patients with suspected recurrence of breast cancer and concluded that FDG-PET/CT can improve staging and therefore affect clinical management in patients with suspected breast cancer recurrence and distant metastatic disease.

In conclusion, FDG-PET or FDG-PET/CT cannot replace SLND and is not recommended for routine lymph node staging [41], nor for staging patients with I, IIA, or IIB breast cancer [50]. Instead, it should be included into the work-up when distant metastases or recurrent breast cancer are suspected [41], for detection of extra-axillary lymph node metastases and distant metastases [26,31], and when lymph nodes are palpable or locally advanced breast cancer is present [51].

### 2.3. Treatment Response Evaluation

Total cure in metastatic breast cancer is rarely achieved; however, with the proper therapy the survival and quality of life of these patients can be extended. Some studies have demonstrated promising results when using FDG-PET for assessing treatment response. For example, Gennari *et al.* [52] showed not only that the decrease of FDG uptake was correlated with a good response to treatment in patients with metastatic disease but also that the response evaluated with FDG-PET could be predictable immediately after the first cycle of chemotherapy [53]. More groups of investigators also supported these conclusions [28,54,55] by stating that the absence of FDG uptake after therapy anticipates better survival rates in patients suffering from metastatic breast cancer. So FDG-PET uptake, measured by SUV should be considered as a prognostic tool for metastatic breast cancer patients [56].

Neoadjuvant chemotherapy (NAC) is commonly used in locally advanced breast cancer patients and is considered to be the standard of care for downstaging the tumor prior to surgery, and significantly improves the prognoses of complete pathologic responders *versus* non-responders [22,57,58]. It has been reported that up to 70% of breast cancer patients treated with NAC have a clinical response; however, only around 25% of these will have a partial or complete response when it is correlated with histopathology [57]. Therefore, the efficacy of NAC can only be determined once the tissue is removed from surgery and histopathologically analyzed, which is considered to be the gold standard [57]. In addition, for early stage breast cancer patients NAC has as its main advantage the ability to increase the use of breast conservation in surgery [59].

**Figure 2:**
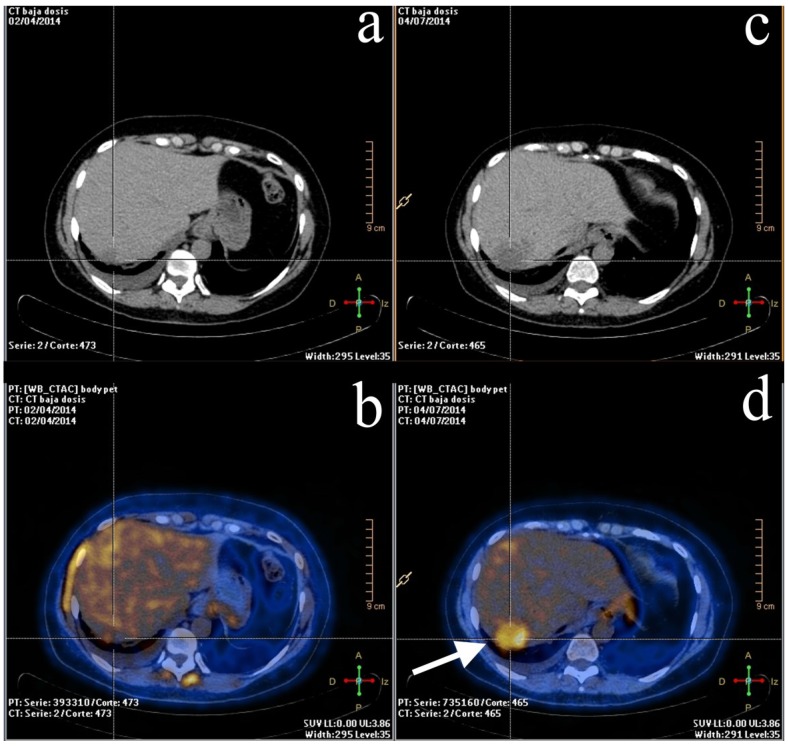
A 53-year-old woman with breast cancer in the course of therapy but with rising biomarkers. The patient was referred for FDG-PET/CT evaluation. *Left column*: baseline PET/CT scan. (**a**) Non-enhanced CT showing a hypodense lesion in the right posterior hepatic lobe; (**b**) PET/CT. Mild metabolism was shown in the lesion, similar to what was found in the background. *Right column*: mid-therapy PET/CT scan. (**c**) Non-enhanced CT showing the same lesion but significantly larger than baseline exam. (**d**) Hypermetabolic lesion with heterogeneous distribution of the radiotracer (arrow) shown on PET/CT, consistent with metastatic liver lesion.

FDG-PET has been proposed as a useful diagnostic tool when there is a necessity for early identification of non-responders (Figure 2). Some studies demonstrated that the mean decrease in FDG uptake after the first cycle of chemotherapy was higher in partial, complete macroscopic, or microscopic lesions [60]. In this study, PET was able to predict complete pathologic response with a sensitivity and specificity of 90% and 74%, respectively. Zucchini *et al.* [24] evaluated metabolic changes with FDG-PET/CT after receiving NAC in early or locally advanced breast cancer patients, showing that early metabolic non-response was always related with histological non-responders and poor prognosis in ER-positive/HER2-negative patients. The authors concluded that FDG-PET/CT could be useful to select patients who might benefit from early therapeutic strategy modifications. A recent study confirmed that same conclusion by assessing the relevance of breast cancer subtypes for monitoring therapy response during NAC with FDG-PET/CT [61]. Response during NAC monitored with FDG-PET/CT was able to predict response in ER-positive/HER2-negative and triple negative subgroup of patients but less accurate in HER2-positive tumors.

A peculiarity when evaluating treatment response with FDG-PET is found in responders after hormonotherapy due to the fact that a transient increase in glucose may occur. This metabolic flare phenomenon is known to be a side effect of anti-estrogen therapy, also associated with increased pain in bone metastases. This effect usually happens 7–10 days after the beginning of the hormonotherapy and is frequently followed by a remission of the disease [57]. Mortimer *et al.* [62] studied this metabolic effect with FDG-PET and 16 alpha-[29]fluoroestradiol-17 beta (FES)-PET in 40 women with biopsy-proved advanced ER-positive breast cancer receiving therapy with tamoxifen and concluded that this flare effect could be an indicator of hormone responsiveness in advanced breast cancer.

One of the challenges that faces not only FDG-PET but other imaging modalities such as MRI, CT, and bone scanning with ^99m^Tc-HDP (or MDP) is to be found in bone metastases. For example, bone scintigraphy, which is performed in many countries, has been part of the work-up in following those patients that have shown false-negatives due to the behavior of some metastases that show a more lytic component than an osteoblastic reaction [63,64]. Also, it has been reported that false-positive results, known as the “flare phenomenon,” can occur after a successful therapy response [65,66].

**Figure 3:**
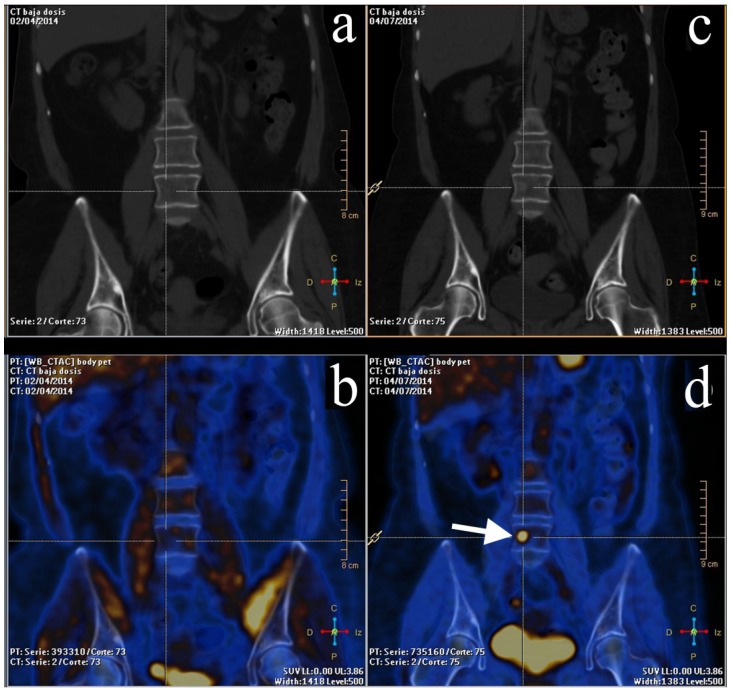
Same patient as Figure 2. *Left column*: baseline PET/CT scan. (**a**,**b**) No abnormality was noticed on CT or PET/CT. *Right column*: mid-therapy PET/CT scan. (**c**) Non-enhanced CT showing an iso-hyperdense rounded lesion in the fourth lumbar vertebrae, called to be as undetermined. (**d**) PET/CT demonstrating a focus of FDG uptake (arrow) in the lumbar vertebrae due to bone metastases.

On the other hand, FDG-PET/CT offers good results in the evaluation of lytic and mixed bone metastases; however, it lacks diagnostic performance when those metastases are found to be the osteoblastic type. Nakai *et al.* [67] had a poor visualization rate in respect to bone metastases with FDG-PET. Other authors compared FDG-PET/CT *versus* CT imaging in the evaluation of early bone marrow metastases in breast cancer, establishing that FDG-PET/CT was more accurate than early detection on bone metastases, improving the staging in up to 15% of their patients. They recommend its use for treatment planning and follow-up (Figure 3) [68].

In comparison with other imaging modalities including CT alone and MRI, FDG-PET/CT demonstrates better diagnostic accuracy [47,69]. CT may be used for the detection of cortical and trabecular bone destruction but has a low sensitivity due to the fact that bone metastases usually start in the bone marrow instead of the cortical tissue, sometimes even more difficult to evaluate when osteoporosis or degenerative changes are also present [64]. Contrariwise, MRI is suitable for detecting bone marrow involvement but not for cortical disruption.

An old PET radiopharmaceutical that has been receiving renewed interest is ^18^F-fluoride. This tracer has shown promise as a good modality for early and accurate detection of bone metastases in many neoplasms. Some suggest that the combination of FDG and fluoride PET imaging allows evaluation of both sclerotic and lytic lesions when assessing treatment response in bone metastases [31]. Damle *et al.* [64] compared the role of FDG-PET/CT, fluoride PET/CT, and bone scintigraphy in the detection of bone metastases in several oncological diseases and suggested that fluoride PET imaging can show bone metastases when progression is clinically suspected or in high risk patients. Also, the authors believed that bone scanning with ^99m^Tc-MDP could be replaced in the near future by fluoride PET in many diseases.

A recently published study compared ^99m^Tc-MDP, FDG-PET/CT, fluoride PET/CT, and MRI for the detection of known osseous metastases, confirming better image quality and superior evaluation performance of skeletal involvement with fluoride PET/CT compared to the other imaging techniques. In addition, FDG-PET/CT showed relevant soft-tissue information that could be helpful in the treatment management of the patients [70].

FDG-PET seems to be useful as a diagnostic tool for evaluating treatment response in locally advanced breast cancer or metastatic disease when change in the treatment strategy is possible. PET should be considered as a valuable imaging modality to predict NAC response in locally advanced breast cancer and chemotherapy in metastatic disease [71,72].

## 3. Dedicated Breast PET Imaging

MM is still the cost-effective imaging modality of choice in standard breast cancer screening procedure; however, it suffers from low specificity in the detection of malignancy and in women with dense breast tissue. Other imaging modalities such as MRI have been tested as an alternative or complement to MM [25]. MRI offers high spatial resolution and excellent soft-tissue contrast, and, with the use of contrast agents, it provides enhancement information. Nonetheless, it is known to have a significant rate of false-positives in benign lesions, and in certain conditions like estrogen-modulation some lesions might be missed due to increased background signal [48]. Also, patients with implanted metal materials (pacemakers, *etc.*) or suffering from claustrophobia are usually excluded from MRI.

Nuclear medicine has been offering many diagnostic tools in primary detection, staging, and the evaluation of treatment response but has recognized limitations, most of them already discussed. Recent developments in breast imaging have been achieved in the last decades based on the challenges that the diversity of breast imaging modalities, including whole-body PET systems, faced. To overcome the known limitations of these imaging techniques, breast-dedicated systems for positron imaging, also known as positron emission mammography (PEM), have been designed. The main benefits of PEM include higher spatial resolution, improved geometric sensitivity with reduced attenuation, shorter imaging time, and the possibility of lowering the radiopharmaceutical dose compared to whole-body PET systems [73].

PEM systems are equipped with two parallel photon detectors, very similar to the configuration used in mammography compressors, which directly correlates with that of MM, facilitating comparison of findings on both modalities. Preliminary studies using this technical approach show a sensitivity around 90%, enabling the detection of small lesions [74]. Other groups have reported 91% sensitivity with 93% specificity [74] in a multicenter study trial. Additionally, the authors exhibited the ability of their PEM system to detect very small lesions (around 2 mm in size) and small foci of ductal carcinoma *in situ* [74]. Higher radiation dose than MM and the difficulty of detecting lesions in the posterior wall were reported in most studies. The variability of FDG uptake in small and less metabolically active tumors, and false-positive imaging findings due to prior biopsy were also observed [16].

When comparing PEM with other imaging modalities besides whole-body PET, a large study of patients who had newly diagnosed early stage breast cancer and who were undergoing conventional imaging, PEM, MRI, and conventional imaging plus PEM, depicted additional disease in 14% of patients, showing better specificity with PEM compared with MRI and thus avoiding unnecessary biopsies [75,76]. Recently, a study showed promising, but preliminary, results of the first high-resolution PEM-guided biopsy study, concluding that this approach is both safe and effective for the sampling of PET-depicted breast lesions [77].

**Figure 4:**
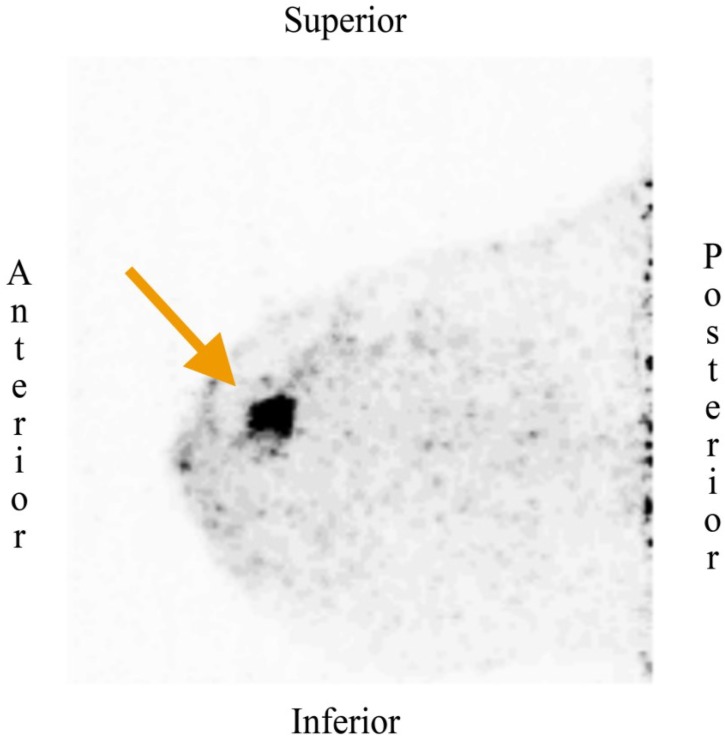
Recently diagnosed breast cancer in a middle-aged woman. The dedicated breast PET scan (MAMMI Breast PET, Oncovision, GEM Imaging SA, Valencia, Spain) shows a focus of intense FDG uptake (arrow) in the upper inner quadrant of the right breast that was proven to be a breast carcinoma. Image courtesy of Dr. Cozar-Santiago and Dr. Ferrer-Rebolleda, Department of Nuclear Medicine, ERESA Medical Group, General University Hospital, Valencia, Spain.

As an alternative to this PEM design, a breast-dedicated high-resolution PET system has recently been developed. This device demonstrates a high detector spatial resolution (approx. 1.5 mm) with fast acquisition times designed in a hanging breast imaging approach. Some of the advantages of this approach are the prone position, which may improve the sensitivity and detection of difficult deep lesions, improved detection of small tumors especially in young women and those with dense breasts, and a portable system that allows imaging in the operation room (Figure 4). A pilot study tried to evaluate the tumor detection and FDG uptake characteristics in 32 patients in stage II and III breast cancer prior to NAC, compared to conventional PET/CT. The conclusion of this study was that this PEM system was able to detect almost all breast lesions, including those close to the thoracic wall [78].

In conclusion, dedicated breast PEM and PET systems seem to be a promising technology that help overcome the limitations of whole-body imaging and could eventually be capable of reliably imaging primary breast neoplasms. Some of the potential roles include detection, local staging, local recurrence, and predicting response to treatment. However, these preliminary results have to be further evaluated with more studies in order to determine the concrete benefits of these devices and their place in the diagnostic algorithm of breast cancer.

## 4. Non-FDG Tracers

The limitations of ^18^F-FDG are well-known as this is not a very specific tracer for breast malignancies. Besides ^18^F-FDG and ^18^F-fluoride, other radiopharmaceuticals have been used in both a pre-clinical and clinical setting.

### 4.1. ^18^F-Fluoromisonidazole

Assuming that around 70% of breast neoplasms are hormone-dependent, overexpressing hormone receptors such as estrogen receptor (ER), endocrine therapy has been established as an important tool to treat ER-positive breast cancer. Conventional anti-estrogen therapy with tamoxifen, for example, has shown worse results than those in postmenopausal patients in early and advanced stage of breast cancer treated with a new generation of hormone treatment with third-generation aromatase inhibitors [79]. However, 30% of ER-positive patients will not properly respond due to primary resistance to hormonal therapy [79].

In this setting, hypoxia is a normal phenomenon that occurs in solid tumors probably because of uncontrolled proliferation and immature blood vessels. Hypoxia-induced factor (HIF), a known biomarker for hypoxia, is known to be associated with tumor propagation, malignant progression, and resistance to treatment [79]. Kurebayashi *et al.* [80] studied the relationship between the growth-promoting effects of estradiol and the growth-inhibitory effects of an anti-estrogen in an ER-positive breast cancer cell line and observed that hypoxia was significantly reduced in both. Another study comparing neoadjuvant letrozole with letrozole plus metronomic cyclophosphamide demonstrated that high levels of HIF-1α were significantly associated with resistance to treatment [81,82].

In PET imaging, radiolabeled hypoxia-avid compounds can be used to evaluate the oxygenation status in experimental or human tumors. ^18^F-labeled fluoromisonidazole, or ^18^F-FMISO, is a nitroimidazole derivative radiopharmaceutical used in research PET/CT. This radiotracer has affinity for hypoxic cells with functional nitroreductase enzymes; therefore it accumulates in activated cells but not in necrotic cells [83]. Several studies have shown superb correlation between the uptake ^18^F-FMISO and oxygenation status of non-small cell lung neoplasms, head and neck cancer, gliomas, and cervical cancer [79].

### 4.2. ^18^F-Fluorothymidine

Among the most commonly used drugs in breast cancer are taxanes. The possibility of imaging early response or resistance of therapy would be an ideal tool for clinicians. ^18^F-labeled fluorothymidine, or ^18^F-FLT, has been proposed as an early molecular imaging biomarker able to evaluate treatment response with taxanes (*i.e.*, doxetacel) [84]. In tumor cells, FLT uptake is regulated by equilibrative nucleoside transporter (ENT-1) and thymidine kinase1 (TK1). TK1 is regulated during cell division transcriptionally in the G1 to S-phase of the cell cycle and hence cellular proliferation [85]. Uptake of FLT is also correlated to Ki-67 labelling index, another proliferation parameter, in breast cancer as shown by Kenny *et al.* [86], and various other cancers demonstrated by Salskov *et al.* [87]. Some studies have presented a strong correlation of FLT uptake with cell proliferation in untreated patients with breast cancer, enabling detection of response as early as 1 week after chemotherapy. Pio *et al*. [88] compared ^18^F-FDG and ^18^F-FLT imaging in 14 patients with newly diagnosed primary or metastatic breast cancer for monitoring and predicting tumor response to chemotherapy. The group concluded that ^18^F-FLT may be more accurate than ^18^F-FDG two weeks after the end of the first course of chemotherapy for predicting longer-term efficacy of chemotherapy for women with breast cancer.

### 4.3. ^18^F-Fluoroestradiol

Levels of ER expression in breast cancer have been shown to have important prognostic information and also to predict the likelihood of a response to hormonal therapy [89]. Immunohistochemistry (IHC) techniques of fixed tissue are now an available diagnostic tool to measure ER expression in newly diagnosed breast cancer. ^18^F-labeled fluoroestradiol or ^18^F-FES is a novel radiopharmaceutical that non-invasively measures ER expression in tumors and has emerged as a valuable method to predict response to hormone therapy in recurrent or metastatic breast cancer patients [90,91,92]. ^18^F-FES has also shown potential to overcome the sampling errors that arise from disease heterogeneity and are associated with *in vitro* assays for cancer. In addition, it simultaneously measures the *in vivo* delivery and binding of estrogens [89]. Level of ^18^F-FES uptake predicts the likelihood of a response to tamoxifen and aromatase inhibitor treatment, as some studies supported [93,94], and could be of use in treatment response assessment in some groups with recurrent or metastatic breast cancer.

## 5. Conclusions

The value of FDG-PET or FDG-PET/CT is mainly limited to staging recurrent or metastatic breast disease and in the evaluation of treatment response. However, recent studies, in addition to technological developments including better performance in sensitivity enabling the depiction of lesions smaller than 1 cm, the possibility of having CT that favors anatomical location, dedicated breast PET systems and the use, mostly in a pre-clinical or research setting, of more specific radiolabeled tracers, open an encouraging future for the usefulness of PET imaging in the management of breast cancer. A combination of some of these radiotracers (*i.e.*, ^18^F-FDG and ^18^F-fluoride) in an individualized approach (patient-by-patient basis) may show a potential inclusion into the algorithm of the different stages of the breast cancer work-up.

## References

[1] Ferlay J., Shin H., Bray F., Forman D., Mathers C., Parkin D. GLOBOCAN 2008 v2.0, Cancer Incidence and Mortality Worldwide: IARC Cancer Base No.10. http://globocan.iarc.fr.

[2] Lacey J.V., Kreimer A.R., Buys S.S., Marcus P.M., Chang S.C., Leitzmann M.F., Hoover R.N., Prorok P.C., Berg C.D., Hartge P. (2009). Breast cancer epidemiology according to recognized breast cancer risk factors in the prostate, lung, colorectal and ovarian (PLCO) cancer screening trial cohort. BMC Cancer.

[3] Iagaru A., Masamed R., Keesara S., Conti P.S. (2007). Breast MRI and ^18^F FDG PET/CT in the management of breast cancer. Ann. Nucl. Med..

[4] Gillies R.J. (2002). *In vivo* molecular imaging. J. Cell. Biochem. Suppl..

[5] James M.L., Gambhir S.S. (2012). A molecular imaging primer: Modalities, imaging agents, and applications. Physiol. Rev..

[6] Aktolun C., Bayhan H., Kir M. (1992). Clinical experience with Tc-99m MIBI imaging in patients with malignant tumors. Preliminary results and comparison with Tl-201. Clin. Nucl. Med..

[7] Campeau R.J., Kronemer K.A., Sutherland C.M. (1992). Concordant uptake of Tc-99m sestamibi and Tl-201 in unsuspected breast tumor. Clin. Nucl. Med..

[8] Del Vecchio S., Salvatore M. (2004). ^99m^Tc-MIBI in the evaluation of breast cancer biology. Eur. J. Nucl. Med. Mol. Imaging.

[9] Brem R.F., Floerke A.C., Rapelyea J.A., Teal C., Kelly T., Mathur V. (2008). Breast-specific gamma imaging as an adjunct imaging modality for the diagnosis of breast cancer. Radiology.

[10] Brem R.F., Fishman M., Rapelyea J.A. (2007). Detection of ductal carcinoma *in situ* with mammography, breast specific gamma imaging, and magnetic resonance imaging: A comparative study. Acad. Radiol..

[11] Weber G. (1977). Enzymology of cancer cells (first of two parts). N. Engl. J. Med..

[12] Wang X., Koch S. (2009). Positron emission tomography/computed tomography potential pitfalls and artifacts. Curr. Probl. Diagn. Radiol..

[13] Shreve P.D., Anzai Y., Wahl R.L. (1999). Pitfalls in oncologic diagnosis with FDG PET imaging: Physiologic and benign variants. Radiographics.

[14] Rosenbaum S.J., Lind T., Antoch G., Bockisch A. (2006). False-positive FDG PET uptake—The role of PET/CT. Eur. Radiol..

[15] Mankoff D.A., Eary J.F., Link J.M., Muzi M., Rajendran J.G., Spence A.M., Krohn K.A. (2007). Tumor-specific positron emission tomography imaging in patients: [^18^F] fluorodeoxyglucose and beyond. Clin. Cancer Res..

[16] Specht J.M., Mankoff D.A. (2012). Advances in molecular imaging for breast cancer detection and characterization. Breast Cancer Res..

[17] Avril N., Menzel M., Dose J., Schelling M., Weber W., Janicke F., Nathrath W., Schwaiger M. (2001). Glucose metabolism of breast cancer assessed by ^18^F-FDG PET: Histologic and immunohistochemical tissue analysis. J. Nucl. Med..

[18] Bos R., van Der Hoeven J.J., van Der Wall E., van Der Groep P., van Diest P.J., Comans E.F., Joshi U., Semenza G.L., Hoekstra O.S., Lammertsma A.A. (2002). Biologic correlates of ^18^fluorodeoxyglucose uptake in human breast cancer measured by positron emission tomography. J. Clin. Oncol..

[19] Scheidhauer K., Walter C., Seemann M.D. (2004). FDG PET and other imaging modalities in the primary diagnosis of suspicious breast lesions. Eur. J. Nucl. Med. Mol. Imaging.

[20] Hodgson N.C., Gulenchyn K.Y. (2008). Is there a role for positron emission tomography in breast cancer staging?. J. Clin. Oncol..

[21] Krammer J., Schnitzer A., Kaiser C.G., Buesing K.A., Sperk E., Brade J., Wasgindt S., Suetterlin M., Schoenberg S.O., Sutton E.J. (2015). ^18^F-FDG PET/CT for initial staging in breast cancer patients—Is there a relevant impact on treatment planning compared to conventional staging modalities?. Eur. Radiol..

[22] Groheux D., Giacchetti S., Delord M., de Roquancourt A., Merlet P., Hamy A.S., Espie M., Hindie E. (2015). Prognostic impact of ^18^F-FDG PET/CT staging and of pathological response to neoadjuvant chemotherapy in triple-negative breast cancer. Eur. J. Nucl. Med. Mol. Imaging.

[23] Liu Y. (2014). Role of FDG PET-CT in evaluation of locoregional nodal disease for initial staging of breast cancer. World J. Clin. Oncol..

[24] Zucchini G., Quercia S., Zamagni C., Santini D., Taffurelli M., Fanti S., Martoni A.A. (2013). Potential utility of early metabolic response by ^18^F-2-fluoro-2-deoxy-d-glucose-positron emission tomography/computed tomography in a selected group of breast cancer patients receiving preoperative chemotherapy. Eur. J. Cancer.

[25] Kalles V., Zografos G.C., Provatopoulou X., Koulocheri D., Gounaris A. (2012). The current status of positron emission mammography in breast cancer diagnosis. Breast Cancer.

[26] Choi Y.J., Shin Y.D., Kang Y.H., Lee M.S., Lee M.K., Cho B.S., Kang Y.J., Park J.S. (2012). The effects of preoperative ^18^F-FDG PET/CT in breast cancer patients in comparison to the conventional imaging study. J. Breast Cancer.

[27] Cochet A., David S., Moodie K., Drummond E., Dutu G., MacManus M., Chua B., Hicks R.J. (2014). The utility of ^18^F-FDG PET/CT for suspected recurrent breast cancer: Impact and prognostic stratification. Cancer Imaging.

[28] Humbert O., Cochet A., Coudert B., Berriolo-Riedinger A., Kanoun S., Brunotte F., Fumoleau P. (2015). Role of positron emission tomography for the monitoring of response to therapy in breast cancer. Oncologist.

[29] Aukema T.S., Vogel W.V., Hoefnagel C.A., Valdes Olmos R.A. (2010). Prevention of brown adipose tissue activation in ^18^F-FDG PET/CT of breast cancer patients receiving neoadjuvant systemic therapy. J. Nucl. Med. Technol..

[30] Cao Q., Hersl J., La H., Smith M., Jenkins J., Goloubeva O., Dilsizian V., Tkaczuk K., Chen W., Jones L. (2014). A pilot study of FDG PET/CT detects a link between brown adipose tissue and breast cancer. BMC Cancer.

[31] Rosen E.L., Eubank W.B., Mankoff D.A. (2007). FDG PET, PET/CT, and breast cancer imaging. Radiographics.

[32] Zhang X., Wu F., Han P. (2014). The role of ^18^F-FDG PET/CT in the diagnosis of breast cancer and lymph nodes metastases and micrometastases may be limited. Hell. J. Nucl. Med..

[33] Kumar R., Chauhan A., Zhuang H., Chandra P., Schnall M., Alavi A. (2006). Clinicopathologic factors associated with false negative FDG-PET in primary breast cancer. Breast Cancer Res. Treat..

[34] Kaida H., Ishibashi M., Fujii T., Kurata S., Ogo E., Tanaka M., Hayabuchi N. (2008). Improved detection of breast cancer on FDG-PET cancer screening using breast positioning device. Ann. Nucl. Med..

[35] Wahl R.L., Siegel B.A., Coleman R.E., Gatsonis C.G., PET study group (2004). Prospective multicenter study of axillary nodal staging by positron emission tomography in breast cancer: A report of the staging breast cancer with PET study group. J. Clin. Oncol..

[36] Gil-Rendo A., Zornoza G., Garcia-Velloso M.J., Regueira F.M., Beorlegui C., Cervera M. (2006). Fluorodeoxyglucose positron emission tomography with sentinel lymph node biopsy for evaluation of axillary involvement in breast cancer. Br. J. Surg..

[37] Kumar R., Zhuang H., Schnall M., Conant E., Damia S., Weinstein S., Chandra P., Czerniecki B., Alavi A. (2006). FDG PET positive lymph nodes are highly predictive of metastasis in breast cancer. Nucl. Med. Commun..

[38] Heudel P., Cimarelli S., Montella A., Bouteille C., Mognetti T. (2010). Value of PET-FDG in primary breast cancer based on histopathological and immunohistochemical prognostic factors. Int. J. Clin. Oncol..

[39] Ekmekcioglu O., Aliyev A., Yilmaz S., Arslan E., Kaya R., Kocael P., Erkan M.E., Halac M., Sonmezoglu K. (2013). Correlation of ^18^F-fluorodeoxyglucose uptake with histopathological prognostic factors in breast carcinoma. Nucl. Med. Commun..

[40] Tatsumi M., Cohade C., Mourtzikos K.A., Fishman E.K., Wahl R.L. (2006). Initial experience with FDG-PET/CT in the evaluation of breast cancer. Eur. J. Nucl. Med. Mol. Imaging.

[41] Fletcher J.W., Djulbegovic B., Soares H.P., Siegel B.A., Lowe V.J., Lyman G.H., Coleman R.E., Wahl R., Paschold J.C., Avril N. (2008). Recommendations on the use of ^18^F-FDG PET in oncology. J. Nucl. Med..

[42] Kim J.Y., Lee S.H., Kim S., Kang T., Bae Y.T. (2014). Tumour ^18^F-FDG uptake on preoperative PET/CT may predict axillary lymph node metastasis in ER-positive/HER2-negative and HER2-positive breast cancer subtypes. Eur. Radiol..

[43] Lovrics P.J., Chen V., Coates G., Cornacchi S.D., Goldsmith C.H., Law C., Levine M.N., Sanders K., Tandan V.R. (2004). A prospective evaluation of positron emission tomography scanning, sentinel lymph node biopsy, and standard axillary dissection for axillary staging in patients with early stage breast cancer. Ann. Surg. Oncol..

[44] Bellon J.R., Livingston R.B., Eubank W.B., Gralow J.R., Ellis G.K., Dunnwald L.K., Mankoff D.A. (2004). Evaluation of the internal mammary lymph nodes by FDG-PET in locally advanced breast cancer (LABC). Am. J. Clin. Oncol..

[45] Riegger C., Koeninger A., Hartung V., Otterbach F., Kimmig R., Forsting M., Bockisch A., Antoch G., Heusner T.A. (2012). Comparison of the diagnostic value of FDG-PET/CT and axillary ultrasound for the detection of lymph node metastases in breast cancer patients. Acta Radiol..

[46] Aukema T.S., Straver M.E., Peeters M.J., Russell N.S., Gilhuijs K.G., Vogel W.V., Rutgers E.J., Olmos R.A. (2010). Detection of extra-axillary lymph node involvement with FDG PET/CT in patients with stage II–III breast cancer. Eur. J. Cancer.

[47] Gaeta C.M., Vercher-Conejero J.L., Sher A.C., Kohan A., Rubbert C., Avril N. (2013). Recurrent and metastatic breast cancer PET, PET/CT, PET/MRI: FDG and new biomarkers. Q. J. Nucl. Med. Mol. Imaging.

[48] Eo J.S., Chun I.K., Paeng J.C., Kang K.W., Lee S.M., Han W., Noh D.Y., Chung J.K., Lee D.S. (2012). Imaging sensitivity of dedicated positron emission mammography in relation to tumor size. Breast.

[49] Dirisamer A., Halpern B.S., Flory D., Wolf F., Beheshti M., Mayerhoefer M.E., Langsteger W. (2010). Integrated contrast-enhanced diagnostic whole-body PET/CT as a first-line restaging modality in patients with suspected metastatic recurrence of breast cancer. Eur. J. Radiol..

[50] NCCN Clinical Practice Guidelines in Oncology. Breast cancer Screening and Diagnosis. V.1.2010. http://demystifyingmedicine.od.nih.gov/DM10/0413-BreastCancer/NCCN%20breast-screening.pdf.

[51] Groheux D., Giacchetti S., Delord M., Hindie E., Vercellino L., Cuvier C., Toubert M.E., Merlet P., Hennequin C., Espie M. (2013). ^18^F-FDG PET/CT in staging patients with locally advanced or inflammatory breast cancer: Comparison to conventional staging. J. Nucl. Med..

[52] Gennari A., Donati S., Salvadori B., Giorgetti A., Salvadori P.A., Sorace O., Puccini G., Pisani P., Poli M., Dani D. (2000). Role of 2-[^18^F]-fluorodeoxyglucose (FDG) positron emission tomography (PET) in the early assessment of response to chemotherapy in metastatic breast cancer patients. Clin. Breast Cancer.

[53] Rousseau C., Devillers A., Campone M., Campion L., Ferrer L., Sagan C., Ricaud M., Bridji B., Kraeber-Bodere F. (2011). FDG PET evaluation of early axillary lymph node response to neoadjuvant chemotherapy in stage II and III breast cancer patients. Eur. J. Nucl. Med. Mol. Imaging.

[54] Dose Schwarz J., Bader M., Jenicke L., Hemminger G., Janicke F., Avril N. (2005). Early prediction of response to chemotherapy in metastatic breast cancer using sequential ^18^F-FDG PET. J. Nucl. Med..

[55] Cachin F., Prince H.M., Hogg A., Ware R.E., Hicks R.J. (2006). Powerful prognostic stratification by [^18^F]fluorodeoxyglucose positron emission tomography in patients with metastatic breast cancer treated with high-dose chemotherapy. J. Clin. Oncol..

[56] Morris P.G., Ulaner G.A., Eaton A., Fazio M., Jhaveri K., Patil S., Evangelista L., Park J.Y., Serna-Tamayo C., Howard J. (2012). Standardized uptake value by positron emission tomography/computed tomography as a prognostic variable in metastatic breast cancer. Cancer.

[57] Avril N.E., Weber W.A. (2005). Monitoring response to treatment in patients utilizing PET. Radiol. Clin. N. Am..

[58] Ooe A., Takahara S., Sumiyoshi K., Yamamoto H., Kawai J., Shiba E. (2013). Relationship between intrinsic subtypes and tumor responses to neoadjuvant chemotherapy in patients with locally advanced breast cancer. Breast Dis..

[59] Redden M.H., Fuhrman G.M. (2013). Neoadjuvant chemotherapy in the treatment of breast cancer. Surg. Clin. N. Am..

[60] Wahl R.L., Zasadny K., Helvie M., Hutchins G.D., Weber B., Cody R. (1993). Metabolic monitoring of breast cancer chemohormonotherapy using positron emission tomography: Initial evaluation. J. Clin. Oncol..

[61] Koolen B.B., Pengel K.E., Wesseling J., Vogel W.V., Vrancken Peeters M.J., Vincent A.D., Gilhuijs K.G., Rodenhuis S., Rutgers E.J., Valdes Olmos R.A. (2013). FDG PET/CT during neoadjuvant chemotherapy may predict response in ER-positive/HER2-negative and triple negative, but not in HER2-positive breast cancer. Breast.

[62] Mortimer J.E., Dehdashti F., Siegel B.A., Trinkaus K., Katzenellenbogen J.A., Welch M.J. (2001). Metabolic flare: Indicator of hormone responsiveness in advanced breast cancer. J. Clin. Oncol..

[63] Blake G.M., Park-Holohan S.J., Cook G.J., Fogelman I. (2001). Quantitative studies of bone with the use of ^18^F-fluoride and ^99m^Tc-methylene diphosphonate. Semin. Nucl. Med..

[64] Damle N.A., Bal C., Bandopadhyaya G.P., Kumar L., Kumar P., Malhotra A., Lata S. (2013). The role of ^18^F-fluoride PET-CT in the detection of bone metastases in patients with breast, lung and prostate carcinoma: A comparison with FDG PET/CT and ^99m^Tc-MDP bone scan. Jpn. J. Radiol..

[65] Biersack H.J., Bender H., Palmedo H. (2004). FDG-PET in monitoring therapy of breast cancer. Eur. J. Nucl. Med. Mol. Imaging.

[66] Tu D.G., Yao W.J., Chang T.W., Chiu N.T., Chen Y.H. (2009). Flare phenomenon in positron emission tomography in a case of breast cancer—A pitfall of positron emission tomography imaging interpretation. Clin. Imaging.

[67] Nakai T., Okuyama C., Kubota T., Yamada K., Ushijima Y., Taniike K., Suzuki T., Nishimura T. (2005). Pitfalls of FDG-PET for the diagnosis of osteoblastic bone metastases in patients with breast cancer. Eur. J. Nucl. Med. Mol. Imaging.

[68] Evangelista L., Panunzio A., Polverosi R., Ferretti A., Chondrogiannis S., Pomerri F., Rubello D., Muzzio P.C. (2012). Early bone marrow metastasis detection: The additional value of FDG-PET/CT *vs.* CT imaging. Biomed. Pharmacother..

[69] Sher A., Vercher-Conejero J.L., Muzic R.F., Avril N., Plecha D. (2014). Positron emission tomography/magnetic resonance imaging of the breast. Semin. Roentgenol..

[70] Iagaru A., Young P., Mittra E., Dick D.W., Herfkens R., Gambhir S.S. (2013). Pilot prospective evaluation of 99mTc-MDP scintigraphy, ^18^F NaF PET/CT, ^18^F FDG PET/CT and whole-body MRI for detection of skeletal metastases. Clin. Nucl. Med..

[71] Facey K., Bradbury I., Laking G., Payne E. (2007). Overview of the clinical effectiveness of positron emission tomography imaging in selected cancers. Health technol. Assess..

[72] Asensio C., Cabrera A., Carreras J., Llamas J., Peñuelas I., Pons F., Richter J. (2009). Proposal by the Spanish Society of Nuclear Medicine (SEMN) for approval of PET radiopharmaceuticals indications via the compassionate use. http://www.semnim.es/media/doc_semnim/SEMN_radiofarmacosPET_2009.pdf.

[73] Koolen B.B., Vogel W.V., Vrancken Peeters M.J., Loo C.E., Rutgers E.J., Valdes Olmos R.A. (2012). Molecular imaging in breast cancer: From whole-body PET/CT to dedicated breast pet. J. Oncol..

[74] Berg W.A., Weinberg I.N., Narayanan D., Lobrano M.E., Ross E., Amodei L., Tafra L., Adler L.P., Uddo J., Stein W. (2006). High-resolution fluorodeoxyglucose positron emission tomography with compression (“positron emission mammography”) is highly accurate in depicting primary breast cancer. Breast J..

[75] Berg W.A., Madsen K.S., Schilling K., Tartar M., Pisano E.D., Larsen L.H., Narayanan D., Ozonoff A., Miller J.P., Kalinyak J.E. (2011). Breast cancer: Comparative effectiveness of positron emission mammography and mr imaging in presurgical planning for the ipsilateral breast. Radiology.

[76] Kalinyak J.E., Berg W.A., Schilling K., Madsen K.S., Narayanan D., Tartar M. (2014). Breast cancer detection using high-resolution breast pet compared to whole-body PET or PET/CT. Eur. J. Nucl. Med. Mol. Imaging.

[77] Kalinyak J.E., Schilling K., Berg W.A., Narayanan D., Mayberry J.P., Rai R., Dupree E.B., Shusterman D.K., Gittleman M.A., Luo W. (2011). PET-guided breast biopsy. Breast J..

[78] Koolen B.B., Aukema T.S., Gonzalez Martinez A.J., Vogel W.V., Caballero Ontanaya L., Vrancken Peeters M.J., Vroonland C.J., Rutgers E.J., Benlloch Baviera J.M., Valdes Olmos R.A. (2013). First clinical experience with a dedicated PET for hanging breast molecular imaging. Q. J. Nucl. Med. Mol. Imaging.

[79] Cheng J., Lei L., Xu J., Sun Y., Zhang Y., Wang X., Pan L., Shao Z., Zhang Y., Liu G. (2013). ^18^F-fluoromisonidazole PET/CT: A potential tool for predicting primary endocrine therapy resistance in breast cancer. J. Nucl. Med..

[80] Kurebayashi J., Otsuki T., Moriya T., Sonoo H. (2001). Hypoxia reduces hormone responsiveness of human breast cancer cells. Jpn. J. Cancer Res..

[81] Generali D., Berruti A., Brizzi M.P., Campo L., Bonardi S., Wigfield S., Bersiga A., Allevi G., Milani M., Aguggini S. (2006). Hypoxia-inducible factor-1alpha expression predicts a poor response to primary chemoendocrine therapy and disease-free survival in primary human breast cancer. Clin. Cancer Res..

[82] Generali D., Buffa F.M., Berruti A., Brizzi M.P., Campo L., Bonardi S., Bersiga A., Allevi G., Milani M., Aguggini S. (2009). Phosphorylated eralpha, HIF-1alpha, and mapk signaling as predictors of primary endocrine treatment response and resistance in patients with breast cancer. J. Clin. Oncol..

[83] Lee S.T., Scott A.M. (2007). Hypoxia positron emission tomography imaging with ^18^F-fluoromisonidazole. Semin. Nucl. Med..

[84] Dittmann H., Jusufoska A., Dohmen B.M., Smyczek-Gargya B., Fersis N., Pritzkow M., Kehlbach R., Vonthein R., Machulla H.J., Bares R. (2009). 3'-deoxy-3'-[^18^F]fluorothymidine (FLT) uptake in breast cancer cells as a measure of proliferation after doxorubicin and docetaxel treatment. Nucl. Med. Biol..

[85] Contractor K.B., Kenny L.M., Stebbing J., Rosso L., Ahmad R., Jacob J., Challapalli A., Turkheimer F., Al-Nahhas A., Sharma R. (2011). [^18^F]-3'deoxy-3'-fluorothymidine positron emission tomography and breast cancer response to docetaxel. Clin. Cancer Res..

[86] Kenny L.M., Vigushin D.M., Al-Nahhas A., Osman S., Luthra S.K., Shousha S., Coombes R.C., Aboagye E.O. (2005). Quantification of cellular proliferation in tumor and normal tissues of patients with breast cancer by [^18^F]fluorothymidine-positron emission tomography imaging: Evaluation of analytical methods. Cancer Res..

[87] Salskov A., Tammisetti V.S., Grierson J., Vesselle H. (2007). FLT: Measuring tumor cell proliferation *in vivo* with positron emission tomography and 3'-deoxy-3'-[^18^F]fluorothymidine. Semin. Nucl. Med..

[88] Pio B.S., Park C.K., Pietras R., Hsueh W.A., Satyamurthy N., Pegram M.D., Czernin J., Phelps M.E., Silverman D.H. (2006). Usefulness of 3'-[F-18]fluoro-3'-deoxythymidine with positron emission tomography in predicting breast cancer response to therapy. Mol. Imaging Biol..

[89] Peterson L.M., Mankoff D.A., Lawton T., Yagle K., Schubert E.K., Stekhova S., Gown A., Link J.M., Tewson T., Krohn K.A. (2008). Quantitative imaging of estrogen receptor expression in breast cancer with pet and ^18^F-fluoroestradiol. J. Nucl. Med..

[90] Kumar P., Mercer J., Doerkson C., Tonkin K., McEwan A.J. (2007). Clinical production, stability studies and PET imaging with 16-alpha-[^18^F]fluoroestradiol ([^18^F]FES) in ER positive breast cancer patients. J. Pharm. Pharm. Sci..

[91] Peterson L.M., Kurland B.F., Link J.M., Schubert E.K., Stekhova S., Linden H.M., Mankoff D.A. (2011). Factors influencing the uptake of ^18^F-fluoroestradiol in patients with estrogen receptor positive breast cancer. Nucl. Med. Biol..

[92] Kenny L.M., Al-Nahhas A., Aboagye E.O. (2011). Novel PET biomarkers for breast cancer imaging. Nucl. Med. Commun..

[93] Mortimer J.E., Dehdashti F., Siegel B.A., Katzenellenbogen J.A., Fracasso P., Welch M.J. (1996). Positron emission tomography with 2-[^18^F]fluoro-2-deoxy-d-glucose and 16alpha-[^18^F]fluoro-17beta-estradiol in breast cancer: Correlation with estrogen receptor status and response to systemic therapy. Clin. Cancer Res..

[94] Linden H.M., Stekhova S.A., Link J.M., Gralow J.R., Livingston R.B., Ellis G.K., Petra P.H., Peterson L.M., Schubert E.K., Dunnwald L.K. (2006). Quantitative fluoroestradiol positron emission tomography imaging predicts response to endocrine treatment in breast cancer. J. Clin. Oncol..

